# Highly variable response to cytotoxic chemotherapy in carcinoma-associated fibroblasts (CAFs) from lung and breast

**DOI:** 10.1186/1471-2407-8-364

**Published:** 2008-12-11

**Authors:** Maike Sonnenberg, Heiko van der Kuip, Silke Haubeiß, Peter Fritz, Werner Schroth, Godehard Friedel, Wolfgang Simon, Thomas E Mürdter, Walter E Aulitzky

**Affiliations:** 1 Dr Margarete Fischer-Bosch Institute of Clinical Pharmacology and University of Tuebingen, Auerbachstr. 112, 70376 Stuttgart, Germany; 2 Department of Diagnostic Medicine, Pathology, Robert Bosch Hospital, Auerbachstr. 110 70376 Stuttgart, Germany; 3 Klinik Schillerhöhe, Department of Thoracic Surgery, Gerlingen, Germany; 4 Department of Gynecology, Robert Bosch Hospital, Auerbachstr 110, 70376 Stuttgart, Germany; 5 2nd Department of Internal Medicine, Oncology and Hematology, Robert Bosch Hospital, Auerbachstr. 110, 70376 Stuttgart, Germany

## Abstract

**Background:**

Carcinoma-associated fibroblasts (CAFs) can promote carcinogenesis and tumor progression. Only limited data on the response of CAFs to chemotherapy and their potential impact on therapy outcome are available. This study was undertaken to analyze the influence of chemotherapy on carcinoma-associated fibroblasts (CAFs) *in vitro *and *in vivo*.

**Methods:**

The *in vivo *response of stromal cells to chemotherapy was investigated in 22 neoadjuvant treated breast tumors on tissue sections before and after chemotherapy. Response to chemotherapy was analyzed *in vitro *in primary cultures of isolated CAFs from 28 human lung and 9 breast cancer tissues. The response was correlated to *Mdm2*, *ERCC1 *and *TP53 *polymorphisms and *TP53 *mutation status. Additionally, the cytotoxic effects were evaluated in an *ex vivo *experiment using cultured tissue slices from 16 lung and 17 breast cancer specimens.

**Results:**

Nine of 22 tumors showed a therapy-dependent reduction of stromal activity. Pathological response of tumor or stroma cells did not correlate with clinical response. Isolated CAFs showed little sensitivity to paclitaxel. In contrast, sensitivity of CAFs to cisplatinum was highly variable with a GI50 ranging from 2.8 to 29.0 μM which is comparable to the range observed in tumor cell lines. No somatic *TP53 *mutation was detected in any of the 28 CAFs from lung cancer tissue. In addition, response to cisplatinum was not significantly associated with the genotype of *TP53 *nor *Mdm2 *and *ERCC1 *polymorphisms. However, we observed a non-significant trend towards decreased sensitivity in the presence of *TP53 *variant genotype. In contrast to the results obtained in isolated cell culture, in tissue slice culture breast cancer CAFs responded to paclitaxel within their microenvironment in the majority of cases (9/14). The opposite was observed in lung cancer tissues: only few CAFs were sensitive to cisplatinum within their microenvironment (2/15) whereas a higher proportion responded to cisplatinum in isolated culture.

**Conclusion:**

Similar to cancer cells, CAF response to chemotherapy is highly variable. Beside significant individual/intrinsic differences the sensitivity of CAFs seems to depend also on the cancer type as well as the microenvironment.

## Background

Carcinomas are complex tissues in which cancer cells interact with their surrounding stromal compartment composed of fibroblasts, infiltrating immune cells, vascular network, and extracellular matrix (ECM) molecules. Cancer cells actively influence their adjacent stroma by producing stroma-modulating growth factors such as platelet-derived growth factor (PDGF), vascular endothelial growth factor (VEGF), basic fibroblast growth factor (bFGF), interleukins, and tumor growth factor beta (TGFbeta) [1,2]. Once activated, tumor fibroblasts (also termed "carcinoma-associated fibroblasts", CAFs) differ from normal fibroblasts [1] and exhibit a distinct gene expression pattern [3]. Various studies have shown that CAFs express a range of growth factors and extracellular matrix remodeling enzymes that modulate proliferation and invasion of tumor cells and tumor angiogenesis [4-7]. In xenograft models it has been demonstrated that CAFs are much more competent in promoting tumor growth than normal fibroblasts [7]. Importantly, these tumor promoting properties of CAFs appear to be partially independent of the presence of tumor cells and are maintained *in vitro *even in the absence of the epithelial cells [7,8]. Both genetic and epigenetic alterations underlying this stable tumor supporting phenotype of CAFs have been suggested [9-12].

The functional activity of CAFs appears to be an important determinant for the clinical behavior of tumors. Chang et al. analyzed expression profiles of serum-activated fibroblasts as models for wound healing fibroblasts and compared them with profiles obtained from different tumors [13]. They identified gene expression patterns similar to that of activated fibroblasts within the gene expression signature of tumors. This "wound healing signature" was predictive for poor overall survival and increased risk of metastasis in breast, lung, and gastric cancers [13,14]. The importance of the functional state of the stromal cell compartment was further supported by a recent work published by Finak et al. [15]. They analyzed the expression profiles of microdissection-purified tumor stroma from 51 primary breast tumors and identified a stroma cell signature as an independent prognostic factor predicting clinical outcome more precisely than signatures from whole tissue [15].

In view of these central roles of CAFs for the biology of cancer it seems likely that CAFs are also important for the survival of tumor cells following treatment with DNA damaging agents. Stromal fibroblasts can influence chemosensitivity of tumor cells indirectly by producing and activating extracellular matrix (ECM) molecules. This activated ECM confers chemoresistance by integrin-mediated adhesion to fibronectin [16,17]. Only limited data on the response of CAFs to chemotherapy and their potential impact on therapy outcome are available. Using a xenograft prostate tumor model, El Hilali et al. demonstrated a chemotherapy induced loss of overall tumor mass without reduction of the total number of tumor cells [18]. In addition, co-culture experiments and Xenograft models demonstrated that the efficacy of chemotherapy-induced cell cycle arrest or senescence in stromal fibroblasts is critical for the sensitivity of the tumor compartment [19]. These observations support the view that the response of stromal cells to toxic stress contributes to resistance or sensitivity of the tumor to chemotherapy and might contribute to clinical outcome. We therefore investigated short-term effects of chemotherapy on tumor stroma *ex vivo *in primary tissues derived from newly diagnosed breast cancer and lung cancer specimens and in isolated CAFs from breast and lung.

## Methods

### Patients

The database of the Robert Bosch Hospital was reviewed to select women with invasive breast carcinomas referred between 2004 and 2006. Sections for 22 patients were available both from pre-treatment biopsies and post-treatment surgery specimens. Patients' characteristics are given in additional file 1. They received neoadjuvant chemotherapy regimens (as also described in additional file 1) followed by surgery. Pathologic diagnosis as well as tumor and stromal cell grading were performed on a tumor tissue sample obtained by a core needle biopsy before treatment and from the same tumor after surgery. Paraffin-embedded tissue sections (3 μm) were stained with hematoxylin and eosin (H&E).

For isolation of primary cancer associated fibroblasts (CAFs) and tissue slice preparation, fresh material from primary breast and lung tumors were obtained from patients newly diagnosed for breast cancer at the Robert Bosch Hospital (n = 17 for tissue culture and n = 9 for culture of isolated CAFs) and for lung cancer at the Klinik Schillerhöhe (n = 16 for tissue culture and n = 28 for CAF culture) immediately after resection. The investigation was approved by the local ethics committee (project number 396/2005V) and informed consent was obtained from the patients.

### Tissue slice preparation and culture

Tissue slice preparation and culture was performed as described previously [20]. For breast cancer tissue cultivation, we used Mammary Epithelial Cell Growth Medium (PromoCell, Heidelberg, Germany). Tissue slices from lung cancer were cultivated in Airway Epithelial Cell Medium (PromoCell, Heidelberg, Germany). Treatment with paclitaxel started 24 hours after preparation of slices for additional 72 hours. After treatment, slices were fixed in buffered formalin and embedded in paraffin for further investigation by immunohistochemistry.

### Isolation, cultivation, and characterization of carcinoma-associated fibroblasts (CAFs)

Tumor tissue was enzymatically digested using a Tissue Disaggregation Buffer (120 mM NaCl, 5.6 mM Glucose, 2.5 mM MgCl_2 _× 6H_2_0, 5.4 mM KCl, 1 mM NaH_2_PO_4_, 20 mM HEPES, pH 7.2) supplemented with collagenase (167 U/ml), DNase (250 U/ml) and protease (0.25 mg/ml) for 90 min at 37°C. The disaggregated tissue was filtered through a 70 μm cell strainer (BD Falcon) and the flow-through was seeded in cell culture flasks. The outgrowing fibroblasts were cultivated with RPMI1640 medium supplemented with 20% FCS.

We analyzed CAFs isolated from 16 breast and lung cancer tissue specimens for fibroblast activation protein (FAP) and epithelial specific antigen (ESA) expression. Fibroblasts in the tumor stroma synthesize FAP, a type II transmembrane protein that functions as a serine protease. FAP is expressed in more than 90% of stromal fibroblasts associated with colon, breast, and lung carcinomas [21]. The expression of FAP and ESA was investigated in CAFs from 16 breast and lung cancers using a FAP antibody (FAP-scFv36) or ESA antibody (biomeda, Plovdiv, Bulgaria) by FACS analysis (data not shown). We further performed karyotype analysis with the first 4 CAFs (data not shown). To evaluate 50% growth inhibition values (GI50), we examined cell viability using MTT assays. For this, 5,000 to 10,000 CAFs were treated with 100, 90, 60, 45, 30, 20, 13.3, 8.9, 5.9, 3, 1.48, 0.74, 0.15, 0.07, 0.01 μM paclitaxel or 100, 50, 25, 12.5, 6.25, 3.13, 1.57, 0.78, 0.39 μM cisplatinum for 48 hr in a 96 well plate. All studies using CAFs were performed within passages 2 to 5. As a reference, we used a tumor cell line panel consisting of 22 cell lines from lung carcinoma, breast carcinoma, ovarian carcinoma, AML, and CML.

Genotyping of *TP53-Arg72Pro *and *ERCC1-118C/T *in CAFs from lung cancer tissue specimens was performed by direct sequencing. Mutation analysis of *TP53 *was done by sequencing the complete *TP53 *cDNA. We isolated RNA from frozen cell pellets using the RNeasy Kit (Qiagen, Hilden, Germany). RNA was reverse transcribed using the RevertAid™ H Minus First Strand cDNA Synthesis Kit from Fermentas (St. Leon-Rot, Germany). The following Primers were used for cDNA amplification (sense: cgtccagggagcaggtag; antisense: ccacaacaaaacaccagtgc) and sequencing (primer 1: cacatgacggaggttgtgag; primer 2: ccacaacaaaacaccagtgc). For genotyping of *ERCC1 *polymorphism, we isolated DNA from frozen pellets using the DNeasy Blood & Tissue Kit from Qiagen (Hilden, Germany) and performed a PCR with the following primer pair: sense: cctcagacctacgccgaata, antisense: gctggtttctgctcataggc.

SNP analysis of *Mdm2-309T/G *was done with a PCR-RFLP-based technique using PCR primers: sense: cgcgggagttcagggtaaag and antisense: actacgcgcagcgttcacac. The PCR product was digested with 5 units of MspA1I (New England Biolabs, Frankfurt a.M., Germany) at 37°C for 16 h and electrophoresed on a 2% agarose gel stained with ethidium bromide (a representative example is shown in additional file 2).

### Pathologic examination and immunohistochemistry

Both, tumor and stromal cell response to neoadjuvant treatment was analyzed by comparing H&E stained tissue sections from corresponding samples before and after chemotherapy. We evaluated the regression grade of the tumor compartment according to Sinn et al. (grade 0: no effect, grade 1: resorption and tumor sclerosis, grade 2: minimally focally invasive residues of 5 mm or smaller, grade 3: only non-invasive tumor residues, grade 4: no viable tumor cell detectable) [22]. Tumors with regression grades 2, 3, or 4 were defined as tumors with pathological response. To estimate the response of the stromal compartment to chemotherapy, we established a grading system consisting of 4 grading types (representative examples for the grading types are shown in additional file 3). Grade 0 represents tumors with less than 10 fibrocytes per high-power field characterized by small, spindle-shaped nuclei. This grading type corresponds to complete inactive stroma with the lowest cellular density in the stromal area. Grade 1 was defined as mostly inactive stroma with more than 10 fibrocytes per high-power-field and 1–3 vesicular cells (fibroblasts or endothelial cells with enlarged vesicular nuclei). Grade 2 is characterized by intermediate reactive stroma with more than 10 fibrocytes and 3–10 vesicular cells per high-power-field. Tumors with the highest cellular density in stromal area were classified as grade 3 (more than 10 fibrocytes and more than 10 vesicular cells per high-power-field). Stromal response was defined as a change from grade 2 or 3 to grade 0 or 1.

Clinical assessment of chemotherapy was done by comparing tumor size before and after therapy. This included both sonography and MRT. A clinical partial response (cPR) was defined as a >50% reduction in the product of the two longest perpendicular tumor dimensions. Patients not achieving a 50% reduction were considered to be clinical non responders (cNR). Patients without any residual tumor were defined as clinical complete responder (cCR).

KI67 (anti-human KI67 Antigen, Clone MIB-1, 1:50, Dako) staining was performed using the Dako Envision Kit on a DakoCytomation Autostainer (both DakoCytomation) according to the manufacturer's manual. TUNEL staining was done in compliance to the manufacturer's manual (ApopTag^® ^Kit S7100, Chemicon, Göttingen, Germany). Immunohistochemical assessment was performed independently by two observers (MS, PF). The percentage of TUNEL and KI67 positive cells was assessed using an ×40 objective. 3–7 randomly selected fields were examined for each slice. Discrepancies were resolved by simultaneous examination using a double headed microscope.

### Statistics

Statistics were performed using GraphPad Prism 4.0 Software (GraphPad Prism Software Incorp., San Diego, CA, USA). Different groups were compared using the Mann-Whitney test. Correlation between tumor cell response and stroma cell response in tissue culture was calculated using Fisher's exact test.

## Results

### Neoadjuvant chemotherapy effects on the stromal compartment of breast cancer *in vivo*

**Figure 1:**
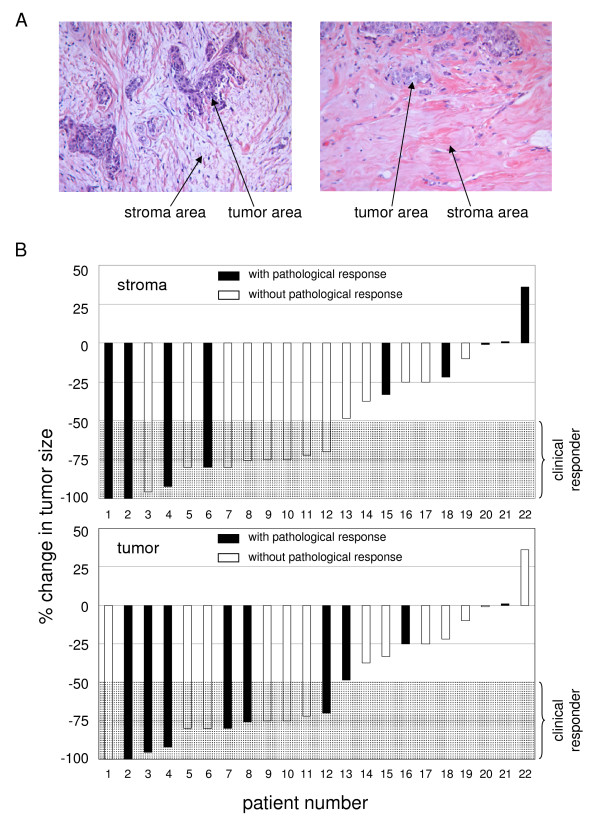
**Stromal cells are target of neoadjuvant chemotherapy *in vivo***. (a) Representative H&E stained sections from corresponding breast cancer samples before (left panel) and after (right panel) chemotherapy. (b) Clinical response and pathological response of stromal cells (upper panel) and tumor cells (lower panel). Before chemotherapy and before surgery, the two longest perpendicular diameters of the tumor were measured either by MRT or sonography. The product of these diameters was used as a measure of tumor size. Partial clinical response (cPR) was defined as reduction of tumor size of more than 50%; tumors with a reduction of less than 50% or induction of size were defined as non responders (cNR). Tumors with pathological stroma response (characterized by a reduction from stromal grade 2 or 3 to grade 0 or 1; upper panel) and tumors characterized by pathological tumor cell response (tumors with regression grade 2, 3, or 4; lower panel) are displayed as black columns.

We investigated the reaction of CAFs on neoadjuvant chemotherapy in a retrospective analysis of breast cancer patients by comparing H&E sections from needle biopsy obtained before treatment with those from the corresponding surgical breast cancer specimen. In 9 of 22 needle biopsies we detected a high cell density in the stromal area before chemotherapy (stroma grade 2 or 3). These 9 cases revealed a significant decrease in stroma cell density after chemotherapy (additional file 1; representative results in Fig. 1a). Pathological tumor cell response was detected in 10 of the 22 cases (regression grade 2, 3, and 4; additional file 1). In 5 cases with stromal response, no change in the tumor cell compartment was observed indicating that stromal response is independent of tumor cell regression (Fisher's exact test: *P *= 0.67; additional file 1).

To determine whether the pathological response of stroma and/or tumor correlates with clinical outcome we compared clinical responders (more than 50% reduction in tumor size) and non-responders (less than 50% reduction or tumor progression). In this small patient cohort, a total of 12 patients showed clinical response including 2 patients with complete tumor regression. Of the 12 tumors with clinical response, 6 showed a significant pathological stromal cell response and 7 responded in their tumor cell compartment with an overlap of 4 cases (Fig. 1b). Three tumors with clinical response showed no pathological response in their stromal nor tumor compartment. These findings suggest that the magnitudes of the reduction for both stroma and tumor cell compartments after cessation of chemotherapy do not correlate with clinical tumor response. However, due to the limited number of patients included in this study, no final conclusion can be drawn.

### Cytotoxic chemotherapy effects on isolated CAFs *in vitro*

We analyzed the acute effects of paclitaxel and cisplatinum on isolated CAFs from primary tumors to evaluate whether CAFs are a direct target of cytotoxic chemotherapy. We isolated CAFs from 9 breast and 28 lung cancer specimens and cultivated them *ex vivo *for up to 10 passages. All CAFs tested were shown to be positive for FAP and negative for the epithelial specific ESA antigen (not shown) indicating that the isolated fibroblasts were highly homogenous with minimal contamination of other cell types. None of the CAFs investigated for karyotype showed any detectable loss or gain of chromosomal material (not shown).

**Figure 2:**
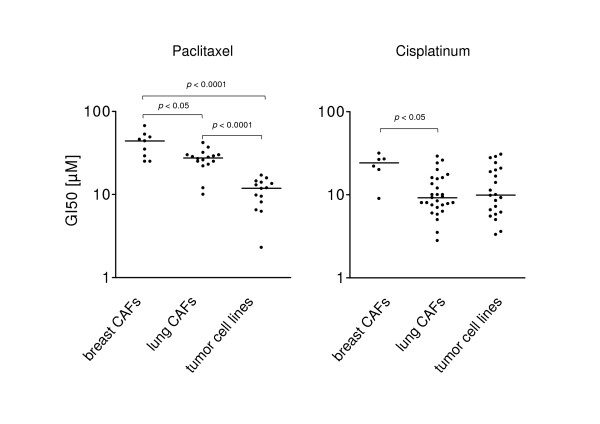
**Sensitivity of CAFs to cytotoxic chemotherapy *in vitro *is highly variable**. Primary CAF cell strains from 9 breast and 28 lung tumors and a panel of 22 tumor cell lines as a reference were cultivated in the presence or absence of different doses of paclitaxel (left panel) or cisplatinum (right panel). Cell growth was measured by means of MTT. Each dot represents the drug concentration to achieve 50% inhibition of cell growth (GI50) in one CAF cell strain or cell line.

Notably, CAFs from lung cancer turned out to grow faster than CAFs isolated from breast tumors (not shown). All CAFs exhibited constant proliferation rates for at least 10 passages (not shown). CAFs were treated with increasing dosages of cisplatinum and paclitaxel for 48 hours (see materials and methods) with cell viability analyzed by means of MTT assay. We compared the 50% growth inhibition (GI50) values from these CAFs to those observed in 22 tumor cell lines. Both CAFs from breast and lung cancer specimens showed a significantly lower sensitivity to paclitaxel than the tumor cell line panel (Fig. 2, left panel). In contrast, the sensitivity to cisplatinum in CAFs was highly variable. CAFs isolated from breast carcinomas were significantly less sensitive than CAFs from lung cancer specimens (GI50 = 22.6 ± 6.8 μM for breast vs. GI50 = 11.4 ± 6.7 μM for lung). The variability of the response of lung CAFs to cisplatinum was comparable to that observed in the cancer cell line panel (GI50 = 13.3 ± 8.7 μM; Fig. 2, right panel). Importantly, the sensitivity to chemotherapeutic drugs did not change significantly when tested in early and late passages (not shown).

### *TP53 *somatic mutations and analysis of *Mdm2*, *ERCC1*, and *TP53 *polymorphisms

The p53 tumor suppressor protein is a central player in the cellular reaction to genotoxic stress [23]. A high frequency of somatic mutations in the *TP53 *gene has been reported in the stromal compartment of breast cancer [11,24]. As we observed a highly variable sensitivity to the DNA damaging agent cisplatinum, we investigated if this variability selectively observed in CAFs from lung cancer is due to genetic changes in genes coding for regulators of DNA damage response. We therefore performed mutation analysis in primary cultured CAFs from lung cancer tissue specimens. No somatic exon mutation was detected in any of the 28 CAFs from lung (not shown).

**Figure 3:**
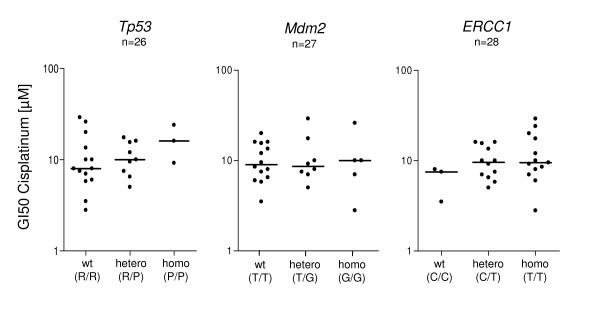
**Sensitivity to cisplatinum is not significantly correlated to polymorphisms in *TP53*, *Mdm2 *and *ERCC1 *in CAFs from lung cancer**. Relationship between cisplatinum sensitivity (GI50 values) and genotype distribution of the polymorphisms *TP53-Arg72Pro *(left panel), *Mdm2-309T/G *(middle panel), and *ERCC1-118C/T *(right panel) in 28 CAF cell strains from lung cancer specimens (solid circles).

The single nucleotide polymorphisms (SNPs) *TP53-Arg72Pro*, *Mdm2-309T/G*, and *ERCC1-118C/T *have been implicated in the clinical response to cisplatinum [25,26]. We therefore tested whether these polymorphisms explain the variable sensitivity to cisplatinum observed in CAFs from lung cancer. As shown in figure 3 (left panel) and additional file 4, the *TP53 *Arg/Pro G>C showed a non-significant trend towards decreased sensitivity in the presence of the variant genotype and neither the *Mdm2 *nor the *ERCC1 *polymorphisms were associated with sensitivity to cisplatinum in these cells (Fig. 3, middle and right panel; additional file 4).

### Impact of cytotoxic chemotherapy on proliferation and cell death of stromal cells within tumor tissue *in vitro*

To evaluate if the sensitivity of CAFs is also determined by the tumor microenvironment, we performed *ex vivo *experiments with tissues from 17 patients with newly diagnosed breast carcinoma and 16 lung cancer patients.

**Figure 4:**
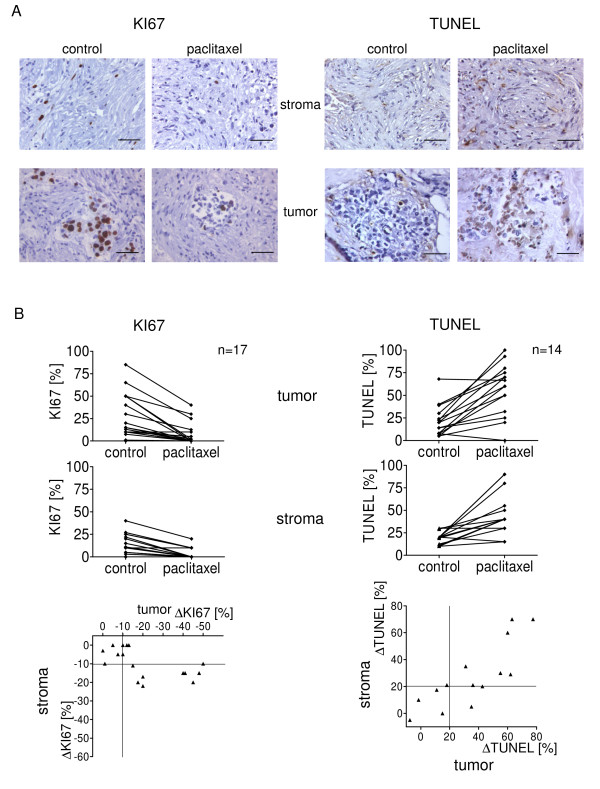
**Cytotoxic chemotherapy targets stromal cells in tissues from breast cancer patients**. Tissue slices from 17 newly diagnosed breast cancer patients were treated with or without paclitaxel for 72 hours, then fixed in formalin and analyzed for proliferation and cell death using KI67 and TUNEL immunohistochemistry, respectively. (a) Representative examples of KI67 (left panel) and TUNEL (right panel) stained sections from tumor tissue slices from one patient incubated with or without paclitaxel. Scale bar, 50 μm. (b) The percentage of KI67 (left histograms) and TUNEL (right histograms) positive cells of both the tumor (upper panel) and the stromal compartment (middle panel) was assessed using an ×40 objective. Three to 7 randomly selected fields were examined for each section. The correlation of tumor and stromal response to paclitaxel regarding changes in proliferation and cell death is shown in the lower panels of the figure. Response to paclitaxel was defined as a reduction of KI67 positive cells by more than 10% (r^2 ^= 0.33; slope = 0.26 ± 0.09; *P *= 0.015) or induction of TUNEL positive cells by more than 20% (r^2 ^= 0.68; slope = 0.76 ± 0.15; *P *< 0.001).

Tissue slices obtained from breast cancer specimens were incubated with or without paclitaxel and analyzed for proliferation and cell death both in stromal and tumor cells using KI67 and TUNEL immunohistochemistry (representative examples are given in Fig. 4a for KI67 (left panel) and for TUNEL (right panel)). Paclitaxel-induced induction of TUNEL positive cells and reduction of KI67 positive cells was observed both in the tumor and stromal cell compartments. Most of the cases showed a reduction in their KI67 index of more than 10% both in their tumor and stromal cells following paclitaxel treatment. Moreover, an increase of TUNEL positive cells of more than 20% was observed in 7 of 14 cases both in tumor and stromal cells. Reduction of both proliferation and increase of cell death was correlated in tumor and stromal cells following paclitaxel treatment (Fig. 4b; *P *= 0.015 and *P *< 0.001, respectively).

**Figure 5:**
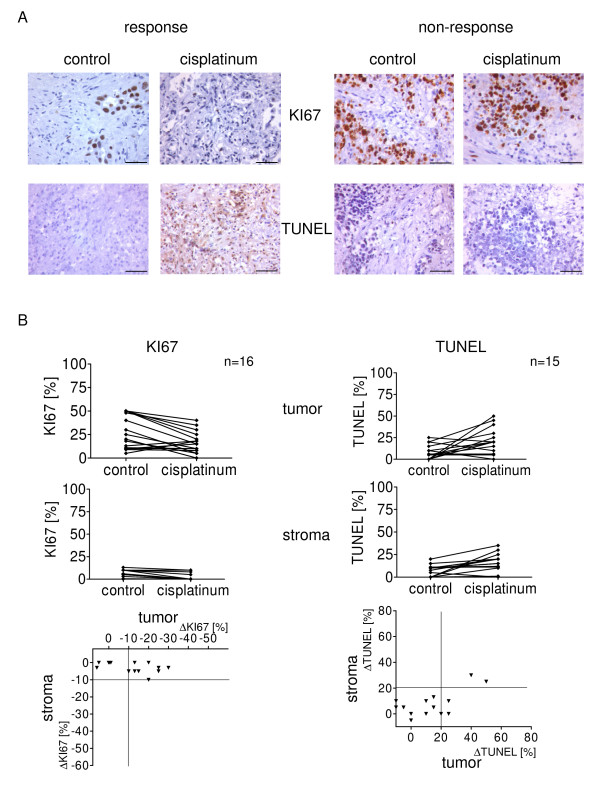
**Effect of cytotoxic chemotherapy on stroma and tumor cells in tissues from lung cancer patients**. Tumor tissue slices from 16 patients with primary lung cancer were incubated with or without cisplatinum for 72 hours, then fixed in formalin and analyzed for proliferation and cell death using KI67 and TUNEL immunohistochemistry. (a) Representative examples of KI67 and TUNEL stained sections from tissues with cellular response (left panel) and non-response (right panel). Scale bar, 50 μm. (b) Percentage of KI67 (left histograms) and TUNEL (right histograms) positive cells of both the tumor (upper panel) and the stromal compartment (middle panel) was assessed using a ×40 objective. Three to 7 randomly selected fields were examined for each section. The correlation of tumor and stromal response to cisplatinum regarding changes in proliferation and cell death is shown in the lower panels of the figure. Response to cisplatinum was defined as a reduction of KI67 positive cells by more than 10% or induction of TUNEL positive cells by more than 20%.

Tissue slices from lung carcinomas were incubated with or without cisplatinum. Figure 5a shows representative examples of tumors with (left panel) or without (right panel) cellular response to cisplatinum as analyzed by KI67 and TUNEL staining. With respect to cell growth, the stromal compartment was less active in lung cancer tissues compared to that observed in breast cancer tissues. Consequently, in lung cancer a cisplatinum-dependent reduction of KI67 positive cells was unique to tumor cells and was observed in 9 of 16 cases (Fig. 5b, left panel). An increase of TUNEL positive cells was observed only in a minority of lung cancer specimens. In 2 cases both the stromal and the tumor cells responded to cisplatinum (Fig. 5b, right panel).

## Discussion

Despite its wide clinical use, the exact mechanisms causing tumor regression after treatment with chemotherapeutic agents are poorly understood. Successful chemotherapy leads to death of malignant cells as well as carcinoma associated fibroblasts and endothelial cells. Whether death of the cancer cell itself is the primary event in the cascade leading to tumor regression remains unclear. In addition, the contribution of the stromal cells to treatment outcome is yet not understood. Our data demonstrate for the first time that not only tumor cells but also CAFs are targeted by cytotoxic chemotherapy both *in vitro *and *in vivo*. Additionally, we found that response to treatment of CAFs from different tumors is highly variable.

Several classification systems have been used to assess the pathological response of tumor cells to neoadjuvant chemotherapy [reviewed in 27]. Anecdotal evidence has also been published for chemotherapy-induced changes of the stromal compartment [28,29]. However, studies focusing on the effects of chemotherapy on stromal cells are not available. In our *in vivo *studies, a stromal response to neoadjuvant chemotherapy was detected in those cases with a reactive stroma before treatment. This stromal regression was independent of tumor cell response implying that both of the tumor compartments can be targeted by chemotherapy despite a lack of response of the other compartment. However, because of the time lag between neoadjuvant chemotherapy cessation and surgery, it is not possible to examine direct effects of chemotherapeutic agents on the different cell compartments in this *in vivo *study setting. We therefore performed *in vitro *experiments with primary cultivated CAFs isolated from newly diagnosed breast and lung carcinomas allowing a comparative study of the cytotoxic response of CAFs to different chemotherapeutic agents.

Orimo et al. demonstrated that isolated fibroblasts from tumors maintain their CAF specific phenotype and their capability to proliferate over at least 10 passages *in vitro *even if epithelial carcinoma cells are not continuously present [7]. In line with this, the CAFs used in the present experiments exhibited a constant proliferation rate over ~10 passages (data not shown). All investigated CAFs were found to be resistant to paclitaxel when compared to a panel of 22 well established cancer cell lines. The CAFs from breast cancer were also mostly resistant to cisplatinum. Only 1 out of 6 tested CAFs from breast cancer showed an intermediate response to this agent. In line with these results, Hawsawi et al. found only 1 of 10 CAFs from breast cancer to be sensitive to cisplatinum [30]. In contrast, we found a remarkable variability of response to cisplatinum in CAFs isolated from lung carcinoma specimens. The GI50 concentrations of cisplatinum ranging from 2.8 to 28 μM were comparable to those obtained in the tumor cell line panel (3.3 to 30.6 μM). Altogether, we conclude that: (1) CAFs respond differently to cisplatinum and paclitaxel; (2) the response of CAFs to cisplatinum shows variability similar to different tumor cell lines; and, (3) CAFs from different organs differ in their response.

Potential causes for the variable response of different CAFs to cisplatinum may be mutations and/or polymorphisms affecting genes involved in DNA damage response mechanisms such as the p53 pathway. Evidence for possible alterations in p53 pathway in CAFs has been provided by several recently published studies. Hawsawi et al. demonstrated that irradiation-induced levels of p53 and p21 are diminished in CAFs when compared to fibroblasts from normal tissues [30]. In addition, several retrospective studies using paraffin-embedded material as source for DNA have reported a high frequency of functional *TP53 *mutations in the microdissected stromal compartment derived from various carcinomas such as breast, ovarian and colon [11,24,31,32]. We did not find any evidence for somatic *TP53 *mutations in the CAFs isolated from 28 lung carcinoma patients included in our study. These results indicate that the presence of *TP53 *mutations in CAFs is limited to certain tumor entities such as breast and colon whereas CAFs from other entities such as lung remain wild type *TP53*. This would imply that the reaction of CAFs to cytotoxic agents and their influence on therapy response are fundamentally different in different organs. However, since many of the studies reporting on high frequencies of somatic mutations in stromal cells have relied on formalin fixed and paraffin embedded material, it can not be ruled out that the identified mutations may be a reflection of methodological limitations. The latter hypothesis is supported by recently published studies by Allinen et al. [3] and Qui et al. [33]. Qui et al. investigated isolated CAFs and frozen tissues and showed that somatic mutations in CAFs from breast and ovarian carcinomas are extremely rare [33]. Allinen et al. separated myofibroblasts and epithelial tumor cells from fresh breast tumor tissues and found no genetic alterations in myofibroblasts whereas numerous chromosomal gains and losses were detected in the epithelial fractions [3]. 

We further examined if the variable sensitivity to cisplatinum observed in CAFs from lung cancer could be attributed to functional polymorphisms in genes critical for cellular response to cisplatinum such as *TP53*, *ERCC1*, and *Mdm2*. It has been shown that cells with the *TP53 Arg/Arg *genotype induce apoptosis significantly better than with *Pro/Pro *genotype [34,35]. The *ERCC1 *Codon 118 polymorphism is associated with different mRNA levels and high levels have been associated with a shorter overall survival for colon carcinoma patients treated with platinum-based chemotherapy [36]. Various studies have shown that the G-allele of the polymorphism *Mdm2-309T/G *in the promoter of the *Mdm2 *gene is associated with high levels of Mdm2 protein and attenuation of the p53 DNA damage response induced by chemotherapeutic agents [37]. Our data show that none of these polymorphisms is significantly correlated with sensitivity to cisplatinum in CAFs from lung cancer. However, we observed a non-significant trend towards decreased sensitivity in the presence of the *TP53 *variant genotype which may be one mechanism contributing to the variable response to cisplatinum in CAFs from lung. Due to the limited number of CAFs included in our study, a final conclusion of potential relevant effects of these polymorphisms on the sensitivity to cytotoxic drugs can not be drawn.

Despite the observation that isolated CAFs retain their cancer promoting phenotype *in vitro *[7], they may behave differently with respect to DNA damage response within the tumor tissue. There is growing evidence that the cellular environment has an important influence on cellular viability. Cell-cell and cell-matrix interactions responsible for this impact have been studied extensively in 2D and 3D *in vitro *culture models [38-40], in spheroid models [41,42] and in co-culture experiments using tumor cell lines and fibroblasts [43]. However, these model systems have some limitations since cell-cell and cell-matrix interactions are extremely complex and specific for each individual tumor *in vivo *[44]. Therefore, we have used a tissue slice culture system which allows the examination of short term effects of chemotherapy on CAFs within their natural environment of the tumor tissue [20]. In tissue cultures from breast carcinomas, paclitaxel-related death of the stromal cell compartment was observed showing an increase of up to 70% apoptotic cells within 72 hours. An interesting finding is that this short term exposure to paclitaxel led to a parallel reaction of both tumor and stromal cells in tissue culture experiments whereas isolated cultured CAFs from breast tumors turned out to be resistant to paclitaxel. These results indicate that within sensitive cancer tissues paclitaxel is highly efficient for compromising the tumor cell compartment and consecutively affecting stroma cells. Within tissue culture, survival of CAFs may be more dependent on supportive factors provided by the individual tumor environment. In contrast, optimized single cell culture conditions for isolated fibroblasts may protect the cells in a more artificial manner. The central role of the microenvironment for response of CAFs to cytotoxic drugs is also demonstrated by our results obtained with lung cancer tissues. In lung cancer, CAFs behave significantly different in cell *versus *tissue culture. The KI67 positive fraction of CAFs in tissues was extremely low whereas isolated CAFs showed a constantly high proliferation. Accordingly, CAFs in tissue turned out to be much less sensitive to cisplatinum when compared to the isolated CAFs. These observations indicate that, in addition to intrinsic factors, the microenvironment determines the sensitivity of CAFs to cytotoxic therapy.

## Conclusion

In conclusion, our *in vivo *and *in vitro *data indicate that stromal reaction is an integral component of tumor response to cytotoxic chemotherapy. Both intrinsic and extrinsic mechanisms influence the variable responses of the stromal compartment to cytotoxic agents. The importance of this variability on the clinical outcome of the carcinoma should be the focus of further investigations.

## Abbreviations

(CAFs): Carcinoma-associated fibroblasts; (GI): growth inhibition; (ECM): extracellular matrix; (FCS): fetal calf serum; (FAP): fibroblast activation protein; (ESA): epithelial specific antigen; (H&E): Haematoxylin & Eosin; (μM): micro molar; (PCR): polymerase chain reaction; (RFLP): Restriction Fragment Length Polymorphism; (cCR): clinical complete responders; (cNR): clinical non responders; (cPR): clinical partial responders; (MRT): magnetic resonance tomography; (SNP): single nucleotide polymorphism; (TUNEL): TdT-mediated dUTP-biotin nick end labeling.

## Competing interests

The authors declare that they have no competing interests.

## Authors' contributions

MS, HvdK, TEM, and WEA designed the study and have been involved in drafting the manuscript. GF and WoS prepared and provided the tumor biological samples. PF and WoS reviewed the patient's histories. MS, SH, and PF participated in the immunohistochemistry studies. WeS and MS performed the genotyping. WeS, TEM, and WEA performed the statistical analysis. All authors discussed and approved the manuscript.

## Pre-publication history

The pre-publication history for this paper can be accessed here:



## Supplementary Material

Additional file 1 **Patients' characteristics.** Table showing patients' characteristics, received neoadjuvant chemotherapy regimens, and response neoadjuvant chemotherapy. Click here for file

Additional file 2 **PCR/RFLP analysis of the *Mdm2-309T/G *polymorphism.** Representative PCR/RFLP patterns for the different *Mdm2 *genotypes: T/T homozygous uncleaved by MspA1I (lanes 1, 4, 5); heterozygous cleaved by MspA1I yielding two bands (lanes 3, 6, 7); G/G homozygous completely cleaved by MspA1I (lanes 2, 8, 9). Click here for file

Additional file 3 **Effect of neoadjuvant chemotherapy on stromal and tumor compartment *in vivo*.** Tumor and stromal cell response to neoadjuvant treatment was analyzed by comparing H&E stained sections from corresponding samples before and after chemotherapy. Tumor cell response was evaluated following the classification of Sinn et al. (22). Tumor stroma types were classified in 4 grading groups. Representative H&E stained slides for each type are shown (upper panel: 400×; lower panel: 1000×). Grade 0 represents tumors with less than 10 fibrocytes per high-power-field characterized by small, spindle-shaped nuclei. This grading type corresponds to complete inactive stroma with the lowest cellular density in the stromal area. Grade 1 was defined as mostly inactive stroma with more than 10 fibrocytes per high-power-field and 1–3 vesicular cells (fibroblasts or endothelial cells with enlarged vesicular nuclei). Grade 2 is characterized by intermediate reactive stroma with more than 10 fibrocytes and 3–10 vesicular cells/high-power-field. Tumors with the highest cellular density in stromal area were classified as grade 3 (more than 10 fibrocytes and more than 10 vesicular cells per high-power-field). Click here for file

Additional file 4 **CAF cell strains from lung carcinomas.** Table showing the GI50 values for cisplatinum and the polymorphisms *TP53-Arg72Pro*, *Mdm2-309T/G*, and *ERCC1-118C/T *in CAF cell strains from lung carcinomas. Click here for file
