# Supplementary material for: Highly variable response to cytotoxic chemotherapy in carcinoma-associated fibroblasts (CAFs) from lung and breast
Source: BMC Cancer. 2008 Dec 11;8:364. doi: 10.1186/1471-2407-8-364 (PMC2626600; doi:10.1186/1471-2407-8-364)
Supplement: Additional file 1 — Patients' characteristics. Table showing patients' characteristics, received neoadjuvant chemotherapy regimens, and response neoadjuvant chemotherapy. [file 1471-2407-8-364-S1.doc]

**Patients´ characteristics**

| Patient  number | age | ypT | G | pN | ER/PR | HER2 | neoadjuvant therapy | clinical response | tumor cell response^#^ | stroma cell response* |
| --- | --- | --- | --- | --- | --- | --- | --- | --- | --- | --- |
| 1 | 42 | 0 | n.d. | 0 | -/- | - | 4xAC, 2xDoc | CR | 4 | - |
| 2 | 58 | 0 | n.d. | 0 | -/- | + | 4xAC, 2xDoc | CR | 1 | - |
| 3 | 46 | 1a | 2 | 1a | +/+ | - | 4xAC, 3xDoc | PR | 4 | - |
| 4 | 66 | 2 | 3 | 0 | -/- | - | 4xAC | PR | 4 | + |
| 5 | 50 | 2 | 3 | 0 | +/+ | + | 3xAC | PR | 1 | + |
| 6 | 44 | 1c | 2 | 0 | +/+ | + | 4xAC, 4xDoc | PR | 1 | + |
| 7 | 45 | 2 | 3 | 1a | +/+ | - | 4xAC, 1xDoc | PR | 4 | + |
| 8 | 43 | 1b | 2 | 1a | +/+ | - | 4xAC, 4xDoc | PR | 2 | + |
| 9 | 52 | 2 | n.d. | n.d. | +/+ | - | 4xAC, 4xDoc | PR | 4 | + |
| 10 | 70 | 4d | 3 | 1 | +/+ | - | Femara, 4xAC, 1xDoc | PR | 2 | - |
| 11 | 65 | 1b | 2 | 2a | +/+ | - | 4xAC, 4xDoc | PR | 1 | - |
| 12 | 44 | 2 | 2 | 1a | +/- | - | 4xAC, 4xDoc | PR | 0 | - |
| 13 | 43 | 2 | 2 | 1a | +/+ | - | 6xTAC | NR | 0 | + |
| 14 | 63 | 1c | 1 | 0 | +/+ | - | 4xAC, 2xDoc | NR | 1 | + |
| 15 | 57 | 3 | 2 | 1a | +/+ | - | 4xAC, 4xDoc | NR | 0 | - |
| 16 | 61 | 3 | 2 | 3 | +/- | + | 4xAC, 4xDoc | NR | 0 | - |
| 17 | 54 | 4d | 2 | 1a | +/+ | - | 4xAC, 4xDoc | NR | 0 | - |
| 18 | 65 | 2 | 2 | 0 | +/+ | + | 4xAC, 2xDoc | NR | 0 | - |
| 19 | 44 | 3 | 2 | 3a | +/+ | - | 4xAC | NR | 1 | + |
| 20 | 43 | 3 | 2 | 3a | +/+ | - | 4xAC, 4xDoc | NR | 2 | - |
| 21 | 57 | 1c | 2 | 0 | +/+ | - | 4xAC, 2xDoc | NR | 3 | - |
| 22 | 47 | 1c | 2 | 0 | +/+ | + | 4xAC | NR | 3 | - |

ypT: tumor size after neoadjuvant chemotherapy; G: tumor grade; pN: pathological nodal status; ER/PR: estrogen receptor/progesterone receptor; HER2: HER2/neu overexpression; CR: complete response; PR: partial response; NR: non response; A: Doxorubicin; C: Cyclophosphamide; Doc: Docetaxel; T: Paclitaxel; ^#^: according to Sinn et. al. (22); *: defined as reduction from grade 2 or 3 to grade 0 or 1
