# Supplementary figures and images for: Highly variable response to cytotoxic chemotherapy in carcinoma-associated fibroblasts (CAFs) from lung and breast
Source: BMC Cancer. 2008 Dec 11;8:364. doi: 10.1186/1471-2407-8-364 (PMC2626600; doi:10.1186/1471-2407-8-364)

1

2

3

4

5

6

7

8

9

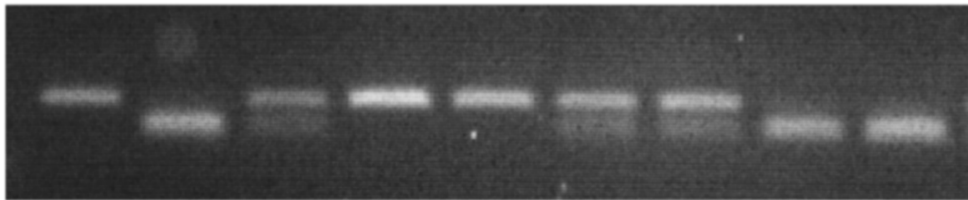

Supplement: Additional file 2 — PCR/RFLP analysis of the Mdm2-309T/G polymorphism. Representative PCR/RFLP patterns for the different Mdm2 genotypes: T/T homozygous uncleaved by MspA1I (lanes 1, 4, 5); heterozygous cleaved by MspA1I yielding two bands (lanes 3, 6, 7); G/G homozygous completely cleaved by MspA1I (lanes 2, 8, 9). [file 1471-2407-8-364-S2.pdf]

0

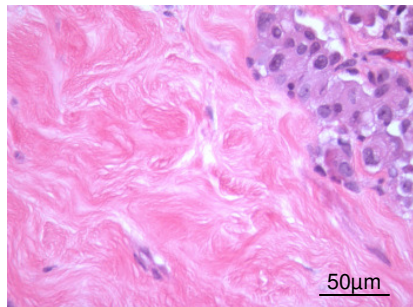

1

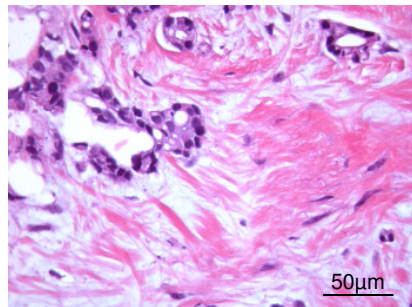

2

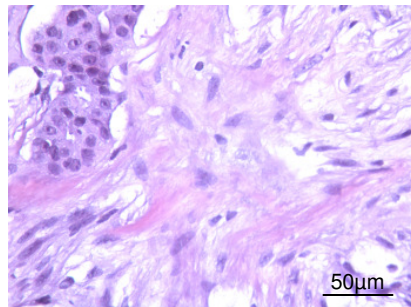

3

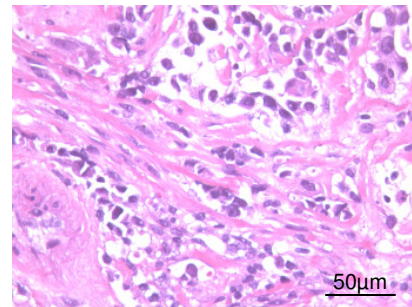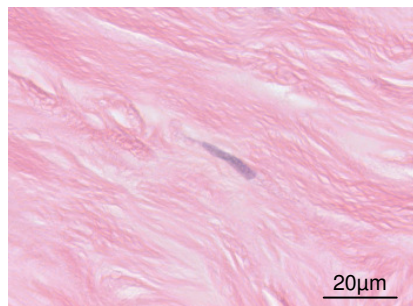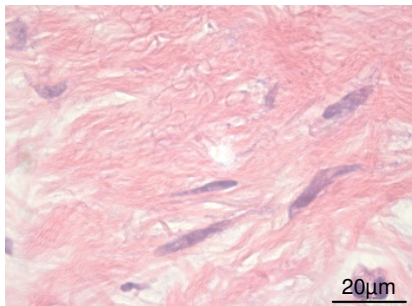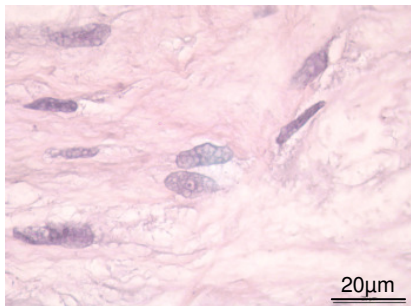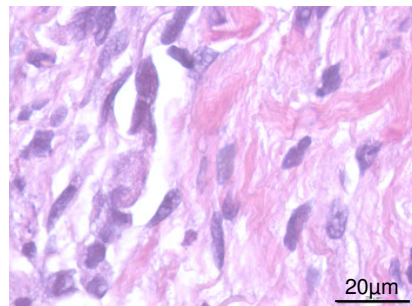

Supplement: Additional file 3 — Effect of neoadjuvant chemotherapy on stromal and tumor compartment in vivo. Tumor and stromal cell response to neoadjuvant treatment was analyzed by comparing H&E stained sections from corresponding samples before and after chemotherapy. Tumor cell response was evaluated following the classification of Sinn et al. (22). Tumor stroma types were classified in 4 grading groups. Representative H&E stained slides for each type are shown (upper panel: 400×; lower panel: 1000×). Grade 0 represents tumors with less than 10 fibrocytes per high-power-field characterized by small, spindle-shaped nuclei. This grading type corresponds to complete inactive stroma with the lowest cellular density in the stromal area. Grade 1 was defined as mostly inactive stroma with more than 10 fibrocytes per high-power-field and 1–3 vesicular cells (fibroblasts or endothelial cells with enlarged vesicular nuclei). Grade 2 is characterized by intermediate reactive stroma with more than 10 fibrocytes and 3–10 vesicular cells/high-power-field. Tumors with the highest cellular density in stromal area were classified as grade 3 (more than 10 fibrocytes and more than 10 vesicular cells per high-power-field). [file 1471-2407-8-364-S3.pdf]
