# Supplementary material for: Highly variable response to cytotoxic chemotherapy in carcinoma-associated fibroblasts (CAFs) from lung and breast
Source: BMC Cancer. 2008 Dec 11;8:364. doi: 10.1186/1471-2407-8-364 (PMC2626600; doi:10.1186/1471-2407-8-364)
Supplement: Additional file 4 — CAF cell strains from lung carcinomas. Table showing the GI50 values for cisplatinum and the polymorphisms TP53-Arg72Pro, Mdm2-309T/G, and ERCC1-118C/T in CAF cell strains from lung carcinomas. [file 1471-2407-8-364-S4.doc]

**CAF cell strains from lung carcinomas**

| **CAFs** | **Cisplatinum GI50 [µM]** | ***Tp53*** | ***Mdm2*** | ***ERCC1*** |
| --- | --- | --- | --- | --- |
|  |  | **[Arg72Pro]** | **[SNP309 T/G]** | **[SNP118 C/T]** |
| 1 | 2.8 | Arg/Arg | G/G | T/T |
| 2 | 3.5 | Arg/Arg | T/T | C/T |
| 3 | 5 | Arg/Pro | T/G | C/T |
| 4 | 5.8 | Arg/Arg | T/T | C/T |
| 5 | 6 | Arg/Arg | T/T | T/T |
| 6 | 6.5 | Arg/Pro | T/T | C/T |
| 7 | 7 | Arg/Arg | G/G | C/T |
| 8 | 7 | n.d. | T/G | T/T |
| 9 | 7.5 | Arg/Arg | T/T | C/C |
| 10 | 7.5 | Arg/Pro | T/G | C/T |
| 11 | 8 | Arg/Arg | T/G | T/T |
| 12 | 8 | Arg/Arg | T/T | C/C |
| 13 | 8.5 | n.d. | T/T | T/T |
| 14 | 9.2 | Pro/Pro | T/G | C/T |
| 15 | 9.5 | Arg/Pro | T/T | T/T |
| 16 | 10 | Arg/Arg | T/G | C/T |
| 17 | 10 | Arg/Arg | G/G | C/T |
| 18 | 10 | Arg/Pro | G/G | T/T |
| 19 | 12 | Arg/Pro | T/T | T/T |
| 20 | 13.5 | Arg/Arg | T/T | C/T |
| 21 | 15.5 | Arg/Pro | T/T | C/T |
| 22 | 16 | Arg/Pro | T/T | C/T |
| 23 | 16 | Pro/Pro | T/T | C/T |
| 24 | 17.5 | Arg/Pro | T/G | T/T |
| 25 | 20 | Arg/Arg | T/T | T/T |
| 26 | 24 | Pro/Pro | n.d. | T/T |
| 27 | 26 | Arg/Arg | G/G | C/C |
| 28 | 29 | Arg/Arg | T/G | T/T |
